# Geographic Differences in Availability and Access to Care Services for Asthma and COPD: Case Study of Vancouver Coastal Health, British Columbia

**DOI:** 10.1155/2024/8019557

**Published:** 2024-10-28

**Authors:** Amelia Choy, Erin M. Shellington, Aneisha Collins-Fairclough, Nardia Strydom, Chris Carlsten

**Affiliations:** ^1^Legacy for Airway Health, Centre for Lung Health, Vancouver Coastal Health Research Institute, Vancouver V5Z 1M9, Canada; ^2^Division of Respiratory Medicine, Department of Medicine, Faculty of Medicine, University of British Columbia, Vancouver V5Z 1M9, Canada; ^3^Department of Family Medicine, Providence Health Care, Vancouver V6Z 1Y6, Canada; ^4^Department of Primary Care, Vancouver Coastal Health Authority, Vancouver, Canada; ^5^Department of Family Practice, Faculty of Medicine, University of British Columbia, Vancouver V6T 1Z3, Canada

## Abstract

**Background:** With a diverse geographic and sociocultural landscape, Vancouver Coastal Health (VCH) Authority (British Columbia, Canada) encompasses both urban and rural regions, providing a case study to examine the delivery of care services for asthma and chronic obstructive pulmonary disease (COPD). To better understand the landscape of care services within VCH, we aimed to (1) identify asthma and COPD care services availability in urban and rural communities and (2) identify where asthma and COPD quality improvement (QI) initiatives were implemented and their implementation-related characteristics.

**Methods:** An environmental scan was conducted to identify asthma and COPD care services provided in VCH communities. A literature review was conducted to determine where VCH asthma and COPD QI initiatives were implemented and identify their implementation-related characteristics. This case study was analysed descriptively and summarised in tables and figures.

**Results:** The environmental scan analysis revealed that specialty outpatient care, pulmonary rehabilitation, respiratory education and clinical smoking cessation services were limited in urban and unavailable in rural VCH communities. Spirometry services were limited in urban and rural VCH communities. Cross-referencing recommendations from asthma and COPD clinical practice guidelines against prevalence data, we estimated that rural VCH communities could provide 0%–23% of required spirometry while urban could provide 40%–75%. Further, of the 16 asthma and COPD QI initiatives identified within 21 papers, none were conducted at rural VCH healthcare sites.

**Conclusion:** Our findings suggest rural VCH communities have lower access to asthma and COPD care services and the limited urban care services were insufficient to make up for this difference. Past asthma and COPD QI initiatives have not apparently translated to care service improvements in rural regions. Future QI initiatives should focus on overcoming barriers to conducting and participating in QI to improve urban and rural regions' access to asthma and COPD care services.

## 1. Introduction

Universal health insurance coverage in countries, including Canada, has enabled access to quality healthcare; however, geography remains a significant access barrier to healthcare [1]. Approximately 19% of Canada's population lives in rural areas with limited access to rural hospitals, healthcare providers and care services [2, 3]. People living in rural parts of Canada disproportionately experience adverse health outcomes compared to their urban counterparts [2, 3]. Rural residents experience a greater burden of noncommunicable diseases, including some of the most common airway diseases, asthma and chronic obstructive pulmonary disease (COPD) [3]. Furthermore, socioeconomic status impacts accessibility in urban regions. Therefore, urban residents' health outcomes should not be overlooked despite their geographic residence [4].

In Canada, urban areas are defined as population centres with a population of at least 1000 and a density of 400 persons per square kilometre [5]. Therefore, rural and remote areas (including rural communities) are all territories outside urban population centres [5, 6]. The low population density in rural communities combined with a diverse geographic and sociocultural landscape makes it challenging to sustain quality healthcare; thus, Canadian residents in rural areas are often required to travel long distances to receive appropriate and necessary care services [7]. For example, rural patients can face expensive travel costs and long wait times to access care [7]. Barriers to accessing urban care services also affect people residing in urban regions, primarily associated with sociodemographic characteristics. For example, racial and ethnic minority groups are more likely to experience long travel times to access care services in urban regions [8].

British Columbia (BC), Canada's third largest province, has its healthcare delivery divided into regional health authorities to serve patients in a geographically diverse province. We use the Vancouver Coastal Health (VCH) Authority as a case study to understand the provision distribution, availability and capacity of asthma and COPD care services in urban and rural regions. In this paper, we use provincial, national and international clinical practice guidelines to define asthma and COPD care services as diagnostic (spirometry), treatment (specialty outpatient care: general respirology, severe asthma and COPD clinics; pulmonary rehabilitation (PR); and clinical smoking cessation) and maintenance (respiratory education) [9–15].

VCH consists of three urban communities, Vancouver, Richmond and North Shore, and five rural communities, Sea-to-Sky Corridor, Sunshine Coast, Powell River, Bella Bella and Bella Coola [16]. See Table 1 for the demographic characteristics of VCH communities. Vancouver is the most populated city in BC that is situated in the western half of the Burrard Peninsula in southwestern Canada. Richmond is a coastal city located south of Vancouver, BC. The North Shore (north of Vancouver) is a community encompassed by three major bodies of water and the peaks of the Coast Mountains to the north. The Coast Mountains and coastline isolate the rural communities beyond the North Shore from the urban regions in VCH. The unique geography of the rural and remote VCH communities makes it difficult for residents to access care services in urban regions. For example, the Sea-to-Sky Corridor is accessible by seaplane, plane, car and daily bus services to urban Vancouver. The Sunshine Coast is only accessible by car and passenger ferry, seaplane and helicopter to the BC mainland. Powell River has limited transportation, with only car and passenger ferry and seaplane options.

Differences in access to urban and rural asthma and COPD care services may contribute to the health disparities between urban and rural residents with asthma or COPD [1]. However, there is limited published evidence on the distribution, availability and capacity of asthma and COPD care services in Canada's urban and rural regions [2]. There is no systematic description of the availability of asthma and COPD care services for residents in VCH communities. In addition, it is unclear how past quality improvement (QI) efforts in VCH may have contributed to the current state of asthma and COPD care services. Therefore, we need to understand where the care services are provided before we can effectively address the health disparities. We conducted an environmental scan and a complementary literature review with VCH as a case study. This involved combining contextual insights with available research evidence to gain a better understanding of the asthma and COPD care services landscape in urban and rural regions. We aimed to (1) identify the availability of asthma and COPD care services in urban and rural VCH communities and (2) determine where past asthma and COPD QI initiatives were conducted within VCH and identify the implementation-related characteristics.

## 2. Materials and Methods

### 2.1. Environmental Scan

An environmental scan was conducted from October 2020 to June 2021 to identify care services for adults living with asthma and COPD across VCH communities. It was conducted during the COVID-19 pandemic when healthcare was generally disrupted. Data on asthma and COPD care services were collected through interviews with VCH key informants (by phone, online Zoom meeting and email), reviewing previous internal VCH environmental scans and an online search of VCH webpages. The interviews were completed under a QI lens and did not require an ethics board's approval [19]. The interview questions are listed in Table 2. Data collected on clinical smoking cessation services refer to behavioural counselling services by referral to a trained healthcare provider [20]. Provincial, national and international clinical practice guidelines do not specify recommendations for clinical smoking cessation, only referring to it as smoking cessation [9–15]. Care services that were excluded were (1) family practice, (2) paediatric care and (3) services in Bella Bella and Bella Coola because there were no available staff to provide data. Patient visit data were excluded because its variable nature made it difficult to obtain. The data obtained included: VCH asthma and COPD prevalence (by region), service locations (defined as rural or urban), presence or absence of asthma and COPD care services, wait times for asthma and COPD care services (if available) and diagnostic testing (spirometry; the number of tests in the previous year(s)). For this paper, we defined spirometry capacity as the number of tests each VCH healthcare site completed in the previous year divided by the COPD prevalence in each VCH community (Table 3). We opted to use only COPD prevalence because (1) guidelines suggest an annual spirometry test for every COPD patient, (2) spirometry test guidelines for asthma patients vary based on severity and we were unable to obtain severity data and (3) diagnosis of asthma or COPD requires a spirometry test and incidence data for asthma and COPD were aggregated across VCH; therefore, we could not obtain community-specific diagnostic test requirements (Table 3). Since COPD prevalence data were only used to calculate spirometry capacities, these capacity calculations were conservative and provided an estimate of spirometry service needs across VCH communities.

### 2.2. Literature Review

A literature review was conducted to identify where past asthma and COPD QI initiatives were implemented in the VCH region and the implementation-related characteristics of these initiatives.

#### 2.2.1. Search Strategy

The search was performed in the CINAHL, Embase, Medline, PsycINFO and Cochrane Library academic databases. Grey literature databases used were the Canadian Best Practices Portal, Canadian Institute for Health Information, CIHR Health Research in Action and Health Evidence. The MeSH terms included were: (‘Asthma' OR ‘Pulmonary Disease, Chronic Obstructive') AND (‘Health Care Delivery, Integrated/AM' OR ‘Health' OR ‘Health Services Research' OR ‘Patient Care Plans' OR ‘Program Evaluation') AND (‘British Columbia').

#### 2.2.2. Screening and Selection

**Figure 1:**
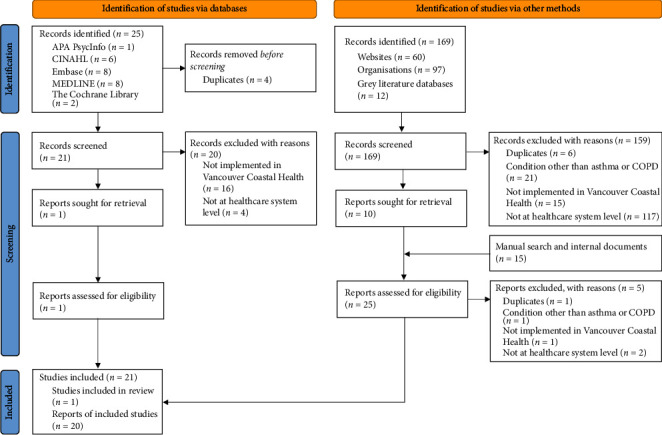
PRISMA flow diagram for the literature review, which included searches of academic databases, grey literature databases and other sources to identify asthma and chronic obstructive pulmonary disease (COPD) quality improvement initiatives in the Vancouver Coastal Health Authority.

Records describing system-level QI initiatives implemented at any VCH healthcare site or (BC) provincial initiative were selected for full-text review. Exclusions were QI initiatives that addressed care services for conditions other than asthma and COPD, implemented in BC regions outside the VCH Authority, were non-English, and completed before 2001 (before VCH existed). Refer to Figure 1 for the PRISMA flowchart of included literature.

#### 2.2.3. Data Extraction

Implementation-related characteristics of the QI initiatives, including: the location and year, roles of the project leads, relevant objectives, outcomes achieved, recommendations related to healthcare access and healthcare system factors interfering with healthcare access, were extracted and summarised descriptively.

### 2.3. Data Analysis

The environmental scan and literature review results were analysed descriptively and summarised in tables and figures.  Table 4 lists VCH hospital and clinic sites and their abbreviated and coded names, where ‘U' in front of the abbreviated name indicates an urban healthcare site and ‘R' indicates a rural healthcare site.

## 3. Results

The environmental scan indicated there were limited to no asthma and COPD care services in rural VCH communities and limited availability and capacity in urban VCH communities. The literature review revealed no asthma and COPD QI initiatives in rural VCH communities (from 2001 to 2021). The long-term impacts of asthma and COPD QI initiatives in urban VCH communities were unknown, with no subsequent QI initiatives published to address system-level challenges and limitations to care access.

### 3.1. Environmental Scan

**Figure 2:**
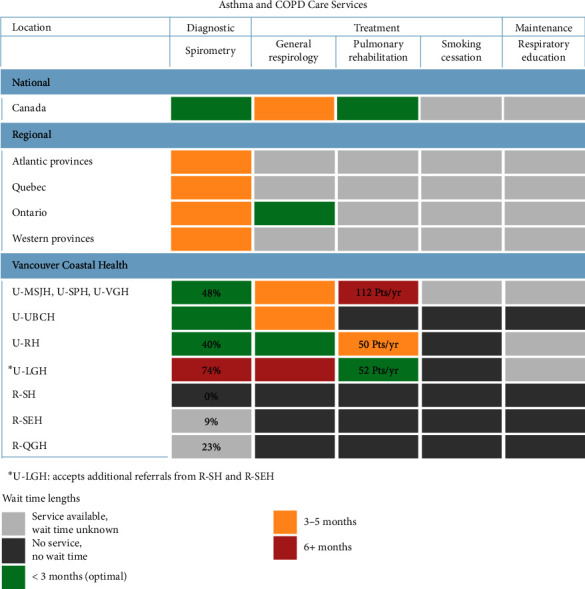
Canadian average wait times for asthma and chronic obstructive pulmonary disease (COPD) care services. Asthma and COPD care services include diagnostic (spirometry), treatment (specialty outpatient care: general respirology; pulmonary rehabilitation; and clinical smoking cessation) and maintenance (respiratory education). Average wait time lengths (in months) were reported at the Canadian national, regional and local Vancouver Coastal Health (VCH) levels [21–24]. Canadian Atlantic provinces include New Brunswick, Nova Scotia, Prince Edward Island and Newfoundland and Labrador. Canadian Western provinces Manitoba, Saskatchewan, Alberta and British Columbia. Wait times of less than 3 months for nonurgent referrals from primary to specialty outpatient care in Canada were optimal as they were considered acceptable by patients [21]. VCH hospital locations have abbreviated code names, where ‘U' in front of the name indicates an urban hospital and ‘R' indicates a rural hospital. Urban hospitals include Mount Saint Joseph's Hospital (U-MSJH), St. Paul's Hospital (U-SPH), Vancouver General Hospital (U-VGH), University of British Columbia (U-UBCH), Richmond Hospital (U-RH) and Lions Gate Hospital (U-LGH). Rural hospitals include Squamish Hospital (R-SH), Sechelt Hospital (R-SEH) and qathet General Hospital (R-QGH). COPD spirometry capacities (as percentages) in VCH are reported at the time of inquiry in 2021 [17]. See Table 3 for the COPD spirometry capacity calculation. COPD spirometry capacities were not obtained for regional Canada and U-UBCH. Note there are only diagnostic services available at U-MSJH and U-VGH is the only VCH hospital that provides clinical smoking cessation services. The number of patients seen annually (Pts/yr) for PR in VCH are reported at the time of inquiry in 2021.

COPD clinical practice guidelines indicate that people living with COPD should have a spirometry test annually [15]. We ascertained (1) COPD prevalence for each VCH community and (2) the number of spirometry tests performed at each VCH healthcare site. As noted in the Materials and Methods section, we did not include asthma prevalence, COPD or asthma incidences due to issues with obtaining these data. Based on the data sources (collected in 2021), we have calculated a conservative estimate that the three rural VCH hospitals provided spirometry tests for 0%, 9% and 23% of COPD patients requiring annual spirometry tests in their local communities. R-SH does not have spirometry services, so patients were referred to U-LGH with available services. U-LGH accepts additional spirometry referrals from R-SEH as well. Accurate documentation for spirometry wait time in rural VCH hospitals was difficult to obtain because these data were not readily available (Figure 2). Five of six urban VCH hospitals could provide spirometry tests for 40%–74% of COPD patients requiring annual spirometry tests in their local communities (Figure 2). As described above, these estimates for spirometry tests include referrals from anywhere (i.e., urban and rural) because the data do not capture where referrals come from. The spirometry capacities for U-UBCH and U-PLC were unknown at the time of inquiry because staff could not provide this data. The data collected for the total number of spirometry tests per year in each VCH community were accurate at the time of collection during the pandemic; however, they may underestimate pre- and postpandemic spirometry service capacities and provide conservative capacities.

At the time of data collection, asthma and COPD treatment and maintenance services, including specialty outpatient care (general respirology, severe asthma and COPD clinics), PR, clinical smoking cessation and respiratory education, were unavailable in rural VCH communities (Figure 2, Table 5). Eight full-time equivalent (FTE) respirologists were available weekly across four outpatient clinics in urban VCH (Table 5). Outreach services for specialty outpatient care (outreach involves respirologist(s) from urban centres travelling to rural areas for patient visits) had been discontinued due to the pandemic, and it was uncertain when or if it would resume. Specialty outpatient care (at U-DCHC, U-PLC, U-TLC, Richmond and North Shore), respiratory therapist (RT)–related services, including respiratory education (at U-DCHC, U-PLC, U-RSCHC, U-TLC, Richmond and North Shore) and PR (at U-SPH, U-VGH, U-RH and U-LGH), and clinical smoking cessation (at U-DCHC, U-TLC, U-VGHSCC) were available but limited in urban VCH communities (Figure 2). Referrals from specialty outpatient care and RT-related services were accepted from rural to urban communities, as is normal in healthcare. For example, PR referrals were accepted from rural VCH communities to U-LGH. The environmental scan data showed that an average of 53 patients per year were seen for PR at each of the urban VCH hospitals (Figure 2). This includes 64 patients annually at U-SPH, 48 at U-VGH, 50 at U-RH and 52 at U-LGH (Figure 2). The wait times for asthma and COPD care services and relevant regional benchmarks in Canada are visually summarised in Figure 2. Aside from the affected outreach services mentioned, there was no indication that the capacities of the other care services had changed from prepandemic.

### 3.2. Literature Review

A total of 16 (11 COPD-related and five asthma-related) QI initiatives with implications in urban VCH from 2009 to 2021 were identified from 21 records (Figure 1; Table 6). Nine out of 16 QI initiatives were conducted across three Vancouver hospitals (U-SPH, U-MSJH and U-VGH), two QI initiatives were conducted in Richmond (U-RH), and one initiative was conducted in the North Shore (U-LGH). Four urban VCH QI initiatives did not state the specific healthcare sites or locations. There were no published QI initiatives that involved rural VCH communities.

Site-specific QI initiatives were led by healthcare and research staff affiliated with an urban VCH hospital. The QI initiatives did not provide a rationale for why their healthcare improvement projects were undertaken in the locations they occurred in; thus, it was assumed for convenience. All QI initiatives were published before the pandemic, so we were unable to determine the impact of the pandemic on QI initiatives.

One out of 16 QI initiatives (2011 COPD Lower Mainland Initiative) aimed to improve access to COPD management services, specifically spirometry access, in the three urban VCH communities (Vancouver, Richmond and North Shore) and the Fraser Health Authority [37, 38, 41]. The initiative developed a universal spirometry requisition and COPD services referral and increased the number of available drop-in spirometry clinics. They increased testing by 50% or more at most sites (the drop-in spirometry clinics located in urban areas, sites unknown), decreased spirometry waitlists to less than 2 weeks at most sites and improved the quality of spirometry interpretations [37, 38, 41]. The evaluation period for this QI initiative was not stated.

Another QI initiative recommended improving COPD care service access in urban VCH communities: ‘Develop a regional strategy to expand community spirometry capacity and behavioural health services' [28]. However, no QI initiatives explicitly recommended addressing asthma and COPD care service availability in rural VCH communities. The BC provincial COPD guidelines stated that spirometry is challenging in rural communities, but they did not identify specific rural regions to target, strategies to address or achievable benchmarks:

‘Timely access to spirometry may be a challenge in rural and remote communities but should remain a reasonable goal. Assuming access to spirometry can occur in a reasonable time frame, a referral to a specialist should not be done before objectively confirming the diagnosis of COPD [11].'

Two urban VCH QI initiatives identified system-level challenges and limitations to accessing care services. The first was a COPD QI initiative in Vancouver, which identified organisational challenges, including patient location and scheduling (i.e., rural patients required to travel long distances to attend in-person appointments) and accessing home care and services away from the hospital [29]. The second was a COPD QI initiative in Vancouver and Richmond that reported disparities in the scale and available resources between a larger academic, tertiary care teaching site (U-VGH) and a community hospital (U-RH) [27]. They noted that U-RH does not have a formal COPD case management program, while there is one currently in place at U-VGH [27]. Although these QI initiatives identified challenges and limitations, we found no published subsequent QI initiatives to address these challenges.

## 4. Discussion

### 4.1. Geography Has Been an Overlooked Factor in Asthma and COPD Care Service Accessibility

Previous research has identified that people in rural and remote communities have limited healthcare access and worse health outcomes than their urban counterparts despite Canada's publicly funded healthcare system [42]. For example, a 2012 Canadian Institute for Health Information (CIHI) report stated that residents in rural, disadvantaged regions experience a higher burden of ambulatory care–sensitive conditions, such as asthma and COPD, than in less disadvantaged urban regions [43]. In the 10 years following the CIHI report, there has been a gap in research (in Canada) to understand how geographic barriers affect healthcare access for people with asthma, COPD or otherwise. Additionally, we found no published QI initiatives for asthma or COPD in rural VCH communities for 20 years (2001 to 2021). The QI initiatives identified in the literature review were in urban regions, and there was no justification for the location described in any of the papers. We assumed that the authors chose the locations because of their affiliation and, thus, convenience. Our results demonstrated differences in the availability of asthma and COPD care services (diagnostic, treatment and maintenance) between urban and rural regions, albeit within a limited geographic portion of one Canadian province. Thus, people with asthma and COPD in rural VCH communities could experience a greater burden in accessing appropriate care services and worse health outcomes than those in urban VCH communities [2, 3]. Our research suggests that geography has been previously overlooked in both research and QI initiatives related to care service availability and access for people with asthma and COPD in VCH.

### 4.2. There Is Limited Access and Availability of Asthma and COPD Care Services in VCH

In VCH, access and availability to asthma and COPD care services are constrained across diagnostic, treatment and maintenance domains. Spirometry, the gold standard for COPD diagnosis, is very limited in availability, with rural VCH communities having almost no capacity (0%–23%) and the spirometry services in urban VCH communities overstretched [15]. Importantly, the capacity for urban sites may be overstated because urban sites accept referrals from rural sites, which likely contributes to the long wait times of more than 6 months at some sites (Figure 2). The limited spirometry availability and delays in access can hinder timely COPD and asthma diagnoses and exacerbate patient health outcomes. Issues with diagnostic service availability and accessibility were acknowledged in the QI initiatives; however, there appear to be limited efforts to improve them and mitigate geographical challenges [11, 28, 37, 38, 41]. Future QI work should focus on (1) understanding factors that impact spirometry capacity, (2) identifying ways to reduce wait times and (3) addressing access issues within the limited resources available, including personnel.

Treatment service availability is similarly inadequate, with no specialty outpatient care, PR and clinical smoking cessation services in rural VCH communities (at the time of the environmental scan) and long wait times of 3 to 6 months to see one of the few respirologists or qualified healthcare providers in urban VCH. It is common for patients from rural VCH communities to be referred to urban specialty outpatient care services. However, they are required to travel for services, use a virtual option (if applicable) or go without. The key informants indicated that the number of PR patients before and during the pandemic was relatively stable; however, all PR programs combined serve an average of 214 patients per year, which would allow access for less than 1% of COPD patients across VCH (Figure 2). Clinical smoking cessation services, a key pillar for COPD care, are only available at three urban healthcare locations in Vancouver (an urban VCH community) to serve the entire VCH region [44]. Two of the three locations only accept referrals from patients who are already clinic patients, and the third had limited availability because of secondment for pandemic-related work (Table 5). Without these qualified individual healthcare providers (i.e., healthcare providers who are Certified Tobacco Educators or addiction specialists), these services would cease to exist. Asthma and COPD treatment services require qualified personnel and space. Without effective efforts to improve appropriate resources, treatment services will presumably remain low, and they may have negative effects on the health outcomes of people with asthma and COPD in both urban and rural communities. There was no apparent focus on improving asthma or COPD treatment services in rural VCH communities, with no involvement of rural healthcare sites in the asthma and COPD QI initiatives. Without effective efforts to improve appropriate resources, treatment services will presumably remain low, and they may have negative effects on patient health outcomes in both urban and rural communities. Although data were collected during the pandemic, key informant interviews indicated that these issues were not new or a result of changes to the health system because of the pandemic but were pervasive, ongoing challenges with respiratory services.

Necessary asthma and COPD maintenance services, such as respiratory education, are confined to VCH urban settings, leaving patients with minimal access and prolonged wait times. Limited respiratory education is provided by nine healthcare providers, all of whom were in urban VCH regions (Table 5). Patients from rural VCH communities can be referred to U-LGH for services. Each healthcare provider has limited clinical time, and the roles of each educator are different, so there is no consistent way to determine their capacity and availability because of how services and personnel are organised (unpublished data). The lack of clarity in services available makes it challenging to determine ways to improve this care service. However, we know that these services would cease to exist without these qualified personnel.

These service availability issues may be more pronounced but not unique for rural patients. Urban asthma and COPD maintenance services were already so limited they did not meet the needs of each urban VCH community, so having additional referrals from rural communities created suboptimal service wait times. Our data demonstrate that these services rely heavily on qualified healthcare providers available to provide appropriate and timely care, creating a precarious situation for necessary care.

### 4.3. Need to Overcome Barriers to Participating in QI to Address Geographic Challenges With Asthma and COPD Care Services

Asthma and COPD QI initiatives were only implemented in urban VCH hospitals where project leads were affiliated. These findings were unsurprising as academic hospitals are in populated urban regions with more services, resources and personnel (compared to rural regions), thus making it more convenient for project leads to conduct QI work [45, 46]. Published QI initiatives were less common in rural and remote healthcare settings. This may be due to physicians in rural regions experiencing compounded challenges in engaging with QI initiatives [47–49].

We postulate that there were intersecting and compounding factors that contribute to the differences in published asthma and COPD QI work between urban and rural healthcare settings, including the type of physician (i.e., specialist or family practitioner) and the focus of their work (i.e., academic appointment or devoted to patient care). Within VCH urban healthcare sites, there were specialist physicians (i.e., respirologists) compared to VCH rural settings that have almost exclusively family practitioners. While family practitioners in rural settings may have academic affiliations, there were often important differences in workload and focus. Unlike family practitioners, specialists with academic appointments may have protected time for research and QI work [50]. These differences in roles and responsibilities between settings may be associated with differences in resources that can benefit urban healthcare sites and negatively impact rural healthcare sites [50]. Urban healthcare sites are more likely to have additional staff (i.e., research staff and more medical trainees) to conduct and publish work. However, family practitioners with academic affiliations in rural settings may have little to no additional staff and resources, so participating in and publishing QI work would be in addition to regular duties and more likely to be considered as ‘side of the desk' passion projects [47].

Our findings complement previous work investigating rural BC physician engagement in QI, including barriers to QI engagement [47]. Markham, Bluman and Lynn identified that time constraints compounded with limited rural physicians and inadequate knowledge of QI principles were barriers. Inadequate knowledge included: identifying areas needed for improvement, developing QI initiatives to address these areas and delivering them effectively [47]. Therefore, future asthma and COPD QI initiatives should address the barriers to QI work in rural settings.

Although all the asthma and COPD QI initiatives we found were implemented in urban VCH communities, they provided limited to no information on their scalability, sustainability and long-term impacts to improve care access in urban and rural settings. The inconsistent long-term sustainability of COPD QI initiatives is consistent with research implemented in other countries with universal healthcare systems [51, 52]. The evaluation periods for the VCH COPD QI initiatives were not clearly stated and no subsequent research was published to address the challenges and limitations reported in the initial QI projects. Also, the urban VCH QI initiatives have not apparently translated to care service improvements in rural regions. We suggest the reasons for the difficulties in implementing and sustaining VCH asthma and COPD QIs were similar to the observed differences in published QI work between urban and rural healthcare settings where considerable time commitment and resources are required for QI project engagement. This further emphasises the need for QI engagement barriers to be addressed to promote the development of asthma and COPD QI initiatives.

### 4.4. Data on Wait Time Benchmarks for Nonurgent Referrals Were Limited but Concerning

We observed limited wait time data on asthma and COPD care services and limited metrics to measure whether reported wait times were considered optimal (Figure 2). This issue was not considered to be pandemic-related, but rather a result of how data are collected and captured by various health teams. Canadian federal benchmarks for wait times for priority procedures such as cataract surgery, hip and knee replacement, radiotherapy and coronary artery bypass exist. However, there is minimal evidence of wait time benchmarks for nonurgent referrals, including care services [53]. Previous literature has recommended a maximum benchmark wait time of 6 months for nonurgent referrals (diagnostic imaging and specialty outpatient care) in Canada [21]. This 6-month recommended benchmark wait time exceeds the maximum 3-month wait time considered acceptable by patients for nonurgent referrals from primary to specialty outpatient care in Canada [21]. In a comparison of specialist wait times in 11 commonwealth countries, Canada reported the longest wait time, with 41% of Canadians waiting over 2 months or more compared to only 5%, 7% and 9% for Switzerland, Germany and the United States, respectively [54]. In BC, there are inadequate incentives to shorten wait times, optimise current systems and improve the quality of care for such services [55]. Insufficient evidence exists regarding appropriate wait time benchmarks for specific nonurgent referrals such as asthma and COPD care services. Without benchmarks, some wait times were longer than 6 months at some sites (Figure 2), combined with limited capacity, suggesting there may be limited ability to reduce wait times.

### 4.5. Limitations

This research provided an initial assessment of the availability and access to asthma and COPD healthcare in urban and rural settings with limited and inadequate quality data that resulted in relatively crude estimates of care services. Much of the data we did have were somewhat dated and, in some aspects, may no longer represent current reality, although we found no particular reason to suspect that things have improved.

This multimethod research was limited as it focussed on health system–level data and did not consider individual care and patient outcomes. We did not measure determinants of health; therefore, we did not determine how much of the variation in access across the urban and rural communities remains after accounting for other determinants [1]. Another limitation is that there could be unpublished asthma and COPD QI initiatives in rural regions that we were not able to find in the literature review and, therefore, not included. The environmental scan relied on our best efforts at informational interviews with relevant health system persons (administrations, managers and providers). Still, the urgency of the pandemic limited the time key informants had to interact with us, potentially impacting the quality and quantity of collected data. Additionally, the data collected from urban VCH locations could not be disaggregated by patient's referral location. Therefore, it is not possible to quantify how many rural patients were referred and were ultimately able to travel to urban centres to receive care. While such travel and care are not infrequent, they pose additional demands on the patient.

## 5. Conclusions and Recommendations

With the global incidence of asthma and COPD increasing, there will be increasing pressure on resources within universal healthcare systems to do more with less [56]. While innovative healthcare provisions exist, this research supports that geographical differences affect the provision and access to asthma and COPD care services in practice. Moreover, the limited comparative data between urban and rural healthcare QI initiatives limit our understanding of the geographic challenges [57]. Future QI initiatives need to consider overcoming barriers to conducting and participating in QI initiatives. This approach could help address healthcare disparities in geographically diverse countries and provide a pathway towards improving access to necessary care services for those living with asthma and COPD in urban and rural communities [58].

To increase access to asthma and COPD care services, we must first understand the current availability and access to care services within and across communities. To do so, we recommend to

1. Improve systematic collection of health system data (i.e., nonurgent referral information and wait time data).
2. Determine and evaluate ongoing needs for better resource allocation of care services at the community level.
3. Encourage researchers to consider both urban and rural healthcare sites for their objectives and outcomes.

## Figures and Tables

**Table 1:** Demographic characteristics of communities in the Vancouver Coastal Health (VCH) authority and the prevalence of asthma and chronic obstructive pulmonary disease (COPD).

Community characteristic	Vancouver Coastal Health (VCH) community (local area profile)					
	Urban			Rural		
	Vancouver (City Centre, Centre North, Northeast, Westside, Midtown, South)	Richmond	North Shore (North Vancouver)	Sea-to-Sky Corridor (Bowen Island/West Vancouver, Howe Sound)	Sunshine Coast	Powell River
VCH hospital site	U-MSJH, U-SPH, U-UBCH, U-VGH	U-RH	U-LGH	R-SH	R-SEH	R-QGH
Median age (years)	39.6	43.6	North Vancouver (City; District Municipality): 42.0; 44.4	Bowen Island: 50.0 West Van: 50.8	56.0	53.2
Average total household income (CAD)	117,300	100,500	North Vancouver (City; District Municipality): 111,800; 170,200	Bowen Island: 166,200 West Van: 212,600	92,200	82,100
Total regional population (in 2017)	676,633	219,273	142,713	91,341	29,390	19,602
Asthma prevalence (number)	11% (74,429)	9% (19,735)	10.5% (14,985)	5% (4567)	13.5% (3968)	13% (2548)
COPD prevalence (number)	6% (40,598)	4% (8771)	5% (7136)	10% (9134)	6.5% (1910)	11% (2156)

*Note:* Where multiple local area profiles existed, a simple average was calculated using regional VCH boundaries [17, 18]. VCH hospital sites have abbreviated code names, where ‘U' in front of the name indicates an urban hospital and ‘R' indicates a rural hospital. Urban hospitals include Mount Saint Joseph's Hospital (U-MSJH), St. Paul's Hospital (U-SPH), Vancouver General Hospital (U-VGH), University of British Columbia (U-UBCH), Richmond Hospital (U-RH) and Lions Gate Hospital (U-LGH). Rural hospitals include Squamish Hospital (R-SH), Sechelt Hospital (R-SEH) and Qathet General Hospital (R-QGH).

**Table 2:** Interview guide to understand the capacity of care services for people living with asthma or chronic obstructive pulmonary disease (COPD) in Vancouver Coastal Health (VCH).

Interview questions
1. Who can access the service? 2. How do patients access the service (referral)? 3. How long is the program/service available for? 4. How many patient-facing full-time equivalents (FTE) per day/week/month? 5. How many patients are seen each day/week/month? a. If there is a waitlist? b. How many people are on it? c. How long is it? 6. How many new patients are seen per clinic/month? a. How many follow-ups occur per clinic/month?

**Table 3:** Calculation and data information used for chronic obstructive pulmonary disease (COPD) spirometry capacity in Vancouver Coastal Health (VCH) [15, 17].

COPD spirometry capacity (%) = (Total spirometry tests per year)/(COPD prevalence)	
Total spirometry tests per year	COPD prevalence
• The spirometry tests per year data were collected from VCH key informants • The total spirometry tests per year for each VCH community was the summation of spirometry tests per year from each VCH hospital and clinic site located in that VCH community • The guideline for annual COPD spirometry was considered when calculating tests per year [15]	• COPD prevalence data for each VCH community were extracted from the 2019 BC Ministry of Health Local Health Area Profiles [17] • COPD prevalence numbers for each VCH community are listed in the last row of Table 1 [17]

**Table 4:** List of Vancouver Coastal Health (VCH) hospital and clinic sites and their abbreviated code names.

Vancouver Coastal Health hospital and clinic site	Abbreviated code name	Description
*Urban (U)*		
Mount Saint Joseph's Hospital	U-MSJH	Urban hospital
St. Paul's Hospital	U-SPH	Urban hospital
University of British Columbia Hospital	U-UBCH	Urban hospital
Vancouver General Hospital	U-VGH	Urban hospital
Richmond Hospital	U-RH	Urban hospital
Lions Gate Hospital	U-LGH	Urban hospital
Pacific Lung Centre	U-PLC	Urban private respirology office
The Lung Centre	U-TLC	Urban private respirology office
Downtown Community Healthcare Centre	U-DCHC	Urban VCH–owned community health centre
Raven Song Community Healthcare Centre	U-RSCHC	Urban VCH–owned community health centre
Vancouver General Hospital Smoking Cessation Clinic	U-VGHSCC	Urban private clinic

*Rural (R)*		
Squamish Hospital	R-SH	Rural hospital
Sechelt Hospital (formally Saint Mary's Hospital)	R-SEH	Rural hospital
Qathet General Hospital (formally Powell River General Hospital)	R-QGH	Rural hospital

**Table 5:** Relevant Vancouver Coastal Health (VCH) healthcare providers available to provide asthma and chronic obstructive pulmonary disease (COPD) maintenance services (at the time of inquiry in 2021).

Vancouver Coastal Health community	Asthma and COPD care services		
	Treatment		Maintenance
	Pulmonary rehabilitation	Clinical smoking cessation	Respiratory education
*Urban (U)*			
Vancouver	0.6 FTE PT; 1 RT, 0.8 FTE RT	1 NP; 1 physician; 1 RN; 2 RTs	2 CREs; 4 RTs
Richmond	0.5 PT; 1 RT	None	1 RT
North Shore	0.8 FTE RT; 0.2 FTE PT; 0.2 FTE PT; 0.2 FTE RN; 0.2 FTE RT	None	0.8 FTE RT

*Rural (R)*			
Sea-to-Sky Corridor	None	None	None
Sunshine Coast	None	None	1 part-time CRE
Powell River	None	None	None

*Note:* Asthma and COPD care services include treatment (pulmonary rehabilitation and clinical smoking cessation) and maintenance (respiratory education). Types of healthcare providers include certified respiratory educator (CRE), nurse practitioner (NP), physician, physiotherapist (PT), registered nurse (RN) and respiratory therapist (RT). Healthcare providers can be part-time or full-time equivalent (FTE).

**Table 6:** Literature review characteristics of the asthma and chronic obstructive pulmonary disease (COPD) quality improvement (QI) initiatives in Vancouver Coastal Health (VCH).

QI initiative	Condition (Asthma/COPD)	Type of literature	Objectives summary	Publication (author, year)	Scope (national/provincial/VCH)	VCH scope (urban/rural)
Canadian Thoracic Society (CTS) guideline focussed updating the management of very mild and mild asthma [12, 13]	Asthma	Academic papers	Update the management of individuals with very mild or mild asthma, currently on as-needed short-acting beta-agonists (PRN SABA) alone or no asthma therapy	Yang et al., 2021a; Yang et al., 2021b	National	Urban
A CTS position statement on the recognition and management of severe asthma [25]	Asthma	Academic paper	Propose a practical approach to distinguish uncontrolled asthma due to inadequate management from severe asthma despite optimal management	FitzGerald et al., 2017	National	Urban
Adult asthma diagnosis and management guideline [9]	Asthma	Guideline	Develop a primary care asthma recognition, diagnosis and management guideline for adults aged ≥ 19 years	Guidelines and Protocols Advisory Committee 2015	Provincial	Urban
Paediatric asthma diagnosis and management guideline [10]	Asthma	Guideline	Develop a primary care asthma diagnosis and management guideline for patients 1–18 years	Guidelines and Protocols Advisory Committee and ChildHealthBC, 2015	Provincial	Urban
Compliance with the Canadian Association of Emergency Physicians' (CAEP) asthma clinical practice guidelines at a tertiary care emergency department [26]	Asthma	Academic paper	Compare asthma management provided at a tertiary care emergency department ED to the management recommended by the CAEP asthma clinical practice guidelines (CPG) and current best practice	Filiatrault et al., 2012	VCH (Vancouver)	Urban
Evaluation of AECOPD management in hospitalised patients [27]	COPD	Academic paper	Determine the proportion of patients admitted to hospital for AECOPD who received treatment adherent to guideline recommendations	Kumar et al., 2020	VCH (Vancouver and Richmond)	Urban
VCH improving COPD community management [28]	COPD	Proposed framework	Provide recommendations on how VCH can improve COPD management.	Farrally et al., 2019	VCH (Vancouver, Richmond and North Shore)	Urban
Emergency department (ED) management of AECOPD: factors predicting readmission [29]	COPD	Academic paper	Compare the readmission rates and patient population demographics of patients visiting the ED for AECOPD and discharged directly from the ED (ED group), to patients visiting the ED and who are then hospitalised for AECOPD (hospitalised group)	Bartels et al., 2018	VCH (Vancouver)	Urban
COPD diagnosis and management guideline [11]	COPD	Guideline	Develop guidelines for the diagnosis and management of COPD adults aged ≥ 19 years	Guidelines and Protocols Advisory Committee, 2017	Provincial	Urban
INSPIRED-adapted Providence COPD outreach program initiative as a part of a quality improvement collaborative (QIC) [30, 31]	COPD	Academic paper	Reduce 30-day readmissions from 17% to 10%, increase advanced care planning (ACP) rates from 20% to 80%, improve quality-of-life scores by 20% and provide individual action plans and advice regarding smoking cessation and medication usage	Rocker et al., 2017; Verma et al., 2018	VCH (Vancouver)	Urban
Impact of individualised care on readmissions after an AECOPD hospitalisation [32]	COPD	Academic paper	Determine the impact of an individualised care package (CP) on early readmission rates following a hospital admission due to AECOPD	Adamson et al., 2016	VCH (Vancouver)	Urban
COPD comprehensive case management program (CCMP) impact [33, 34]	COPD	Academic paper	Determine CCMP efficacy in reducing length of stay (LOS) and risk of COPD hospital (re)admissions	Alshabanat et al., 2015; Alshabanat et al., 2017	VCH (Vancouver)	Urban
Variations in AECOPD management [35, 36]	COPD	Academic paper	Assess AECOPD management at the times of admission and discharge	Sandhu et al., 2013; Vozoris, 2013	VCH (Vancouver)	Urban
COPD lower mainland initiative [37, 38]	COPD	Project charter, academic abstracts only	Streamline, standardise and improve COPD management across the BC lower mainland in acute and primary care	Toplak & FitzGerald, 2010; Toplak et al., 2011	VCH (Vancouver and North Shore) and Fraser Health	Urban
BC health sector information management/information technology (IM/IT) strategy [39]	COPD	Strategy document	Create an IM/IT strategy as an alignment guide for each BC healthcare delivery organisation to develop of their own IM/IT plans	BC eHealth Strategy Council, 2009	Provincial	Urban
Risk factors and outcomes associated with AECOPD requiring hospitalisation [40]	COPD	Academic paper	Determine the rates of hospital readmissions for AECOPD among a cohort of patients previously hospitalised with the same diagnosis and to identify risk factors associated with recurrent hospital admissions for AECOPD	Bahadori et al., 2009	VCH (Vancouver)	Urban

*Note:* An acute exacerbation of chronic obstructive pulmonary disease is abbreviated as AECOPD.

## Data Availability

The qualitative and quantitative data used to support the findings of this study are included within the article.
